# Editorial: Genetic and Epigenetic Mechanisms Underpinning Vulnerability to Developing Psychiatric Disorders

**DOI:** 10.3389/fpsyt.2022.917198

**Published:** 2022-06-13

**Authors:** Noèlia Fernández-Castillo, Elena Martín-García

**Affiliations:** ^1^Departament de Genètica, Microbiologia i Estadística, Facultat de Biologia, Universitat de Barcelona, Catalonia, Spain; ^2^Centro de Investigación Biomédica en Red de Enfermedades Raras (CIBERER), Catalonia, Spain; ^3^Institut de Biomedicina de la Universitat de Barcelona (IBUB), Catalonia, Spain; ^4^Institut de Recerca Sant Joan de Déu (IR-SJD), Esplugues de Llobregat, Barcelona, Spain; ^5^Laboratory of Neuropharmacology-Neurophar, Department of Medicine and Life Sciences, Universitat Pompeu Fabra (UPF), Barcelona, Spain; ^6^Hospital del Mar Medical Research Institute (IMIM), Barcelona, Spain

**Keywords:** behavioral genetics, epigenetics, vulnerability, psychiatric disorders, multifactorial, animal models

The study of genetic and epigenetic mechanisms underlying vulnerability to developing psychiatric disorders has obtained significant outcomes from the behavioral genetics area, providing increased knowledge of individual differences in behavior. Classical research in behavioral genetics with humans was applied in studies of twins, family investigations, and adoption. These studies produced a significant advancement in behavioral genetics, and studies of twins and adoption pointed out that the environment and the interaction between genes and the environment are essential. In addition, molecular genetics has confirmed that the susceptibility to psychiatric disorders is heritable, polygenic, and complex (1). Thus, psychiatric disorders have a complex multifactorial pattern of inheritance (Figure 1). This multifactorial genetic model explains how the interaction between multiple gene networks and environmental factors strongly impacts brain function early during development and later throughout adulthood, influencing behavior (2). Hence, genes are not unique direct triggers of psychiatric disorders but contribute to confer risk for pathological behavior development, accounting for a fraction of total variation (2). An interesting open question is why some individuals are vulnerable to developing a psychiatric disease while others are resilient to certain environmental risk factors, even if their access is not prevented (3).

**Figure 1:**
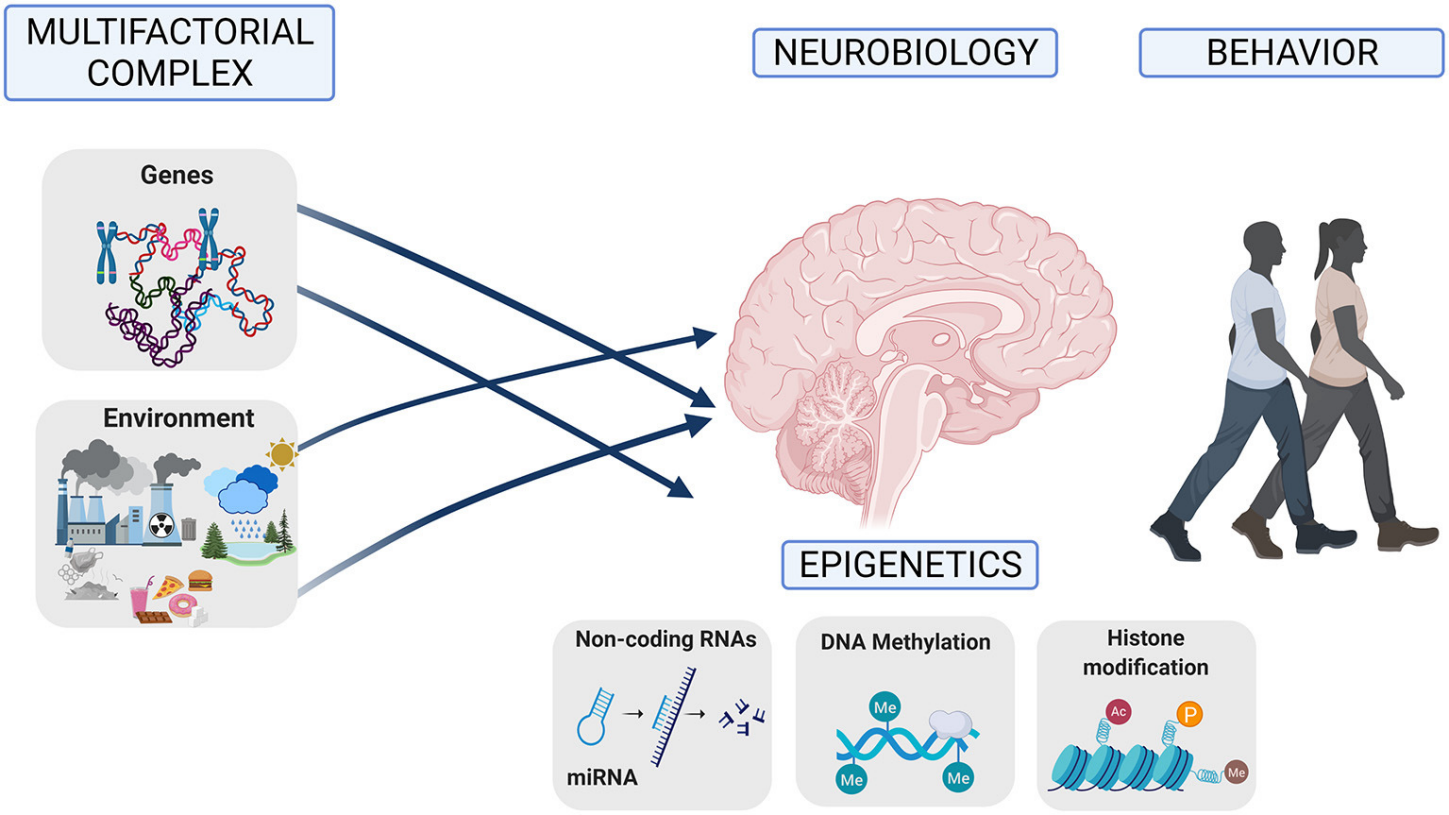
Psychiatric disorders have a complex multifactorial origin with an interaction between multiple genes and multiple environments that influence neurobiology and modify epigenetics during development and adulthood, influencing behavior.

To answer this question, research in psychiatric disorders has dramatically improved thanks to the use of new omic technologies, which allowed hypothesis-free approaches and have impacted the understanding of these disorders and their underlying mechanisms. Articles published in this Research Topics focus on the relevance of using genomic and epigenomic approaches to disentangle the genetic and epigenetic mechanisms underpinning vulnerability to developing psychiatric disorders. In a review article, Singh et al. highlight the contribution of postzygotic somatic mutations to neurodevelopmental and mental disorders, particularly schizophrenia. Postzygotic mutations, which can be detected nowadays with novel genomic and epigenomic technologies, are relatively common in the brain and have been reported in patients with neurodevelopmental disorders. Spencer and Kisby (4) made a commentary article to this review agreeing with their analysis and arguing that these somatic mutations or epimutations may be sufficient to develop a neurodegenerative disorder. Pol-Fuster et al. applied a system genomic approach to identify rare genetic variants involved in psychosis in a family with several members affected. They identified a rare missense variant in the *MACF1* gene and two rare CNVs affecting the genes *CNTN6* and *CDH13* that segregated with psychosis in this family. Liu et al. also performed whole-exome sequencing identifying novel variants in *PGM3* in patients with idiopathic focal epilepsy. Odintsova et al. show the value of combining different omics information for predicting models. For a multi-omics prediction model, the authors combined polygenic scores with methylation scores for smoking status, prenatal maternal exposure, birth weight, and BMI. They found that methylation scores could predict current and former smoking, prenatal maternal smoking, and BMI.

In addition, transcriptomic studies have contributed to understanding the mechanisms involved in psychiatric disorders by directly assessing gene expression alterations and pathways. Using brain samples would be ideal, but unfortunately, this tissue cannot be accessed in patients, and only *post-mortem* samples can be used, which are very difficult to obtain. On the other hand, investigating gene expression alterations in more accessible tissues, such as blood, has become a helpful approach. Bountress et al. investigated differences in gene expression associated with post-stress syndrome disorder (PTSD) and trauma exposure (TE). Peripheral blood mononuclear cells identified 283 genes differentially expressed between PTSD and TE and 7 gene modules enriched in pathways such as focal adhesion or neuroactive ligand-receptor interaction. Zhang et al. used data from *post-mortem* brain samples of patients to identify relevant genes and pathways for bipolar disorders. Three modules of differentially expressed genes were associated with bipolar disorder, with 4 genes identified as hub genes: *NOTCH1, POMC, NGF*, and *DRD2*, being *NOTCH1* replicated in an independent sample. Bipolar disorder has also been associated with an increased prevalence of minor physical anomalies. The brain and the skin share a common origin since they are derived from the ectoderm. Thus, minor physical anomalies may be physical markers of aberrant neurodevelopment. In the work of Csulak and coauthors, it has been demonstrated for the first time that there is an increased prevalence of minor physical anomalies among the first-degree unaffected relatives of bipolar I patients. The study supports the concept that minor physical anomalies can be endophenotypic markers of bipolar I affective disorder (Csulak et al.). The endophenotypes reported were minor physical anomalies in the eye, head, mouth, and trunk regions that were found to be prevalent among the relatives of bipolar I patients compared to normal controls. The individual analyses showed that one minor malformation (sole crease) and one phenogenetic variant (high arched palate) were also more prevalent in the relative group compared to the normal control group (Csulak et al.). It is important to remark that the endophenotype conception of bipolar disorder signifies an essential method in exploring the pathogenesis of the disease. Gottesman (5) described this concept of endophenotype as an intermediate phenotype that fills the gap between genes and diseases.

Candidate gene studies were more frequently used in the past, before the advent of omic technologies, which showed that larger clinical samples were required for obtaining reliable findings in association studies. In this Research Topics, some studies have evaluated the contribution of genetic variants in particular genes. For instance, Shang et al. meta-analyzed previous studies testing the association with an obsessive-compulsive disorder of a single nucleotide polymorphism (SNP) in *BDNF*, an important neurotrophic factor. They found negative results, but gender-specific analyses showed a nominal association in females. Likewise, Komatsu et al. investigated an SNP in the *OLIG2* gene, involved in oligodendrocyte functions, associated with self-schema, a susceptibility trait factor for major depressive disorder. In addition, Tao et al. found a nominal association between temporal lobe epilepsy and an SNP in *DTNBP2*, a gene related to schizophrenia. Finally, Chen et al. found that patients with the first psychosis episode had higher complement components C3 and C4 levels in serum and found that a SNP in the C4 gene was associated.

On the other hand, animal models have helped us understand the role of critical genes in behavioral genetics. For example, Rivero et al. article demonstrates that the haploinsufficiency of the attention-deficit/hyperactivity disorder risk gene *St3gal3* in mice causes alterations in cognition and expression of genes involved in myelination and sialylation. Indeep, the authors found gender differences because male *St3gal3* heterozygous mice showed cognitive deficits, while female heterozygous animals displayed increased activity and cognitive control compared to their wild-type littermates. In addition, expression of several markers implicated in oligodendrogenesis, myelin formation, protein sialylation, and cell adhesion/synaptic target glycoproteins of *ST3GAL3* in a brain region- and sex-specific manner were found. In another article on this issue, the gender differences were highlighted by the work of Reever and colleagues. Their work showed that maternal immune activation during gestation has a prolonged and sex-dependent impact on the transcriptome of the amygdala in pigs (Keever et al.). Specifically, they observed differential gene expression associated with neurodevelopmental illnesses, including schizophrenia and autism spectrum disorders. In schizophrenia, other authors using animal models to study the genetic basis of psychiatric disorders revealed that *IL15R*α KO mice exhibit altered expression of multiple pathways, including lipid biosynthesis and metabolism in the central nervous system (He et al.).

Finally, an interesting view of genomic variation and the paradox of mental illness can be found in this Research Topics (Gualtieri). In this hypothesis and theory article, Gualtieri raises the question of how disorders such as autism and schizophrenia persist and are high heritable, although they confer low reproductive success. The article integrates different perspectives such as heritability and reproductive success and a throughout the view of the contribution of genomic variability at different levels, bringing a novel view to the genetics of psychiatric disorders.

This Research Topic has contributed to a better understanding that psychiatric disorders are complex multifactorial disorders in which nature and nurture interact. Nurture is the personal experience of the interactions with the environment as a raising background, whereas genetic factors define nature. Furthermore, in animal models, nurture has been translationally modeled using procedures that include childhood separation after birth or stress induction (6). Also, the study of the influence of genetic factors in animals elucidated critical landscapes, and it is an evolving field with a promising future to untangle how genetic susceptibility affects the neurobiological substrates of psychiatric disorders. In summary, the new advances in understanding the complex mechanisms involved in the epigenetic control of gene expression and the novel technological tools to manipulate these mechanisms will allow us to understand the ability of environmental influences to alter gene expression and their impact on the progress of the psychiatric disorders. Furthermore, identifying these epigenetic factors opens new opportunities to define accurate biomarkers of vulnerability and resilience to psychiatric disorders and may open pioneering beneficial perspectives that are still unemployed.

## Author Contributions

Both authors listed have made a substantial, direct, and intellectual contribution to the work and approved it for publication.

## Funding

This work was supported by Plan Nacional Sobre Drogas of the Spanish Ministry of Health (#PNSD-2019I006) and by European Union NextGenerationEU/PRTR, the Spanish Ministry of Science and Innovation – MICIN, and the Research State Agency – AEI, through Plan de Recuperación, Transformación y Resiliencia funding program and the ERA-NET neuron translational research projects on neurodevelopmental disorders (MCIN/AEI/UE - PCI2021-122073-2A/10.13039/501100011033) to EM-G. This work was also supported by Plan Nacional Sobre Drogas of the Spanish Ministry of Health (#PNSD-2020I042) to NF-C. Figures are created with BioRender.com.

## Conflict of Interest

The authors declare that the research was conducted in the absence of any commercial or financial relationships that could be construed as a potential conflict of interest.

## Publisher's Note

All claims expressed in this article are solely those of the authors and do not necessarily represent those of their affiliated organizations, or those of the publisher, the editors and the reviewers. Any product that may be evaluated in this article, or claim that may be made by its manufacturer, is not guaranteed or endorsed by the publisher.
